# *TBX6, LHX1* and copy number variations in the complex genetics of Müllerian aplasia

**DOI:** 10.1186/1750-1172-8-125

**Published:** 2013-08-16

**Authors:** Maria Sandbacka, Hannele Laivuori, Érika Freitas, Mervi Halttunen, Varpu Jokimaa, Laure Morin-Papunen, Carla Rosenberg, Kristiina Aittomäki

**Affiliations:** 1 Folkhälsan Institute of Genetics, Helsinki, Finland; 2 Department of Medical Genetics, Haartman Institute, University of Helsinki, Helsinki, Finland; 3 Department of Obstetrics and Gynecology, Helsinki University Central Hospital, Helsinki, Finland; 4 Department of Genetics and Evolutionary Biology, Institute of Biosciences, University of São Paulo, São Paulo, Brazil; 5 Department of Obstetrics and Gynecology, University of Turku, Turku, Finland; 6 Department of Obstetrics and Gynecology, Oulu University Hospital, Oulu, Finland; 7 Department of Clinical Genetics, HUSLAB, Helsinki University Central Hospital, Helsinki, Finland

## Abstract

**Background:**

Müllerian aplasia (MA) is a congenital disorder of the female reproductive tract with absence of uterus and vagina with paramount impact on a woman’s life. Despite intense research, no major genes have been found to explain the complex genetic etiology.

**Methods and Results:**

We have used several genetic methods to study 112 patients with MA. aCGH identified CNVs in 8/50 patients (16%), including 16p11.2 and 17q12 deletions previously associated with MA. Subsequently, another four patients were shown to carry the ~0.53 Mb deletion in 16p11.2. More importantly, sequencing of *TBX6,* residing within 16p11.2, revealed two patients carrying a splice site mutation*.* Two previously reported *TBX6* variants in exon 4 and 6 were shown to have a significantly higher frequency in patients (8% and 5%, respectively) than in controls (2% each). We also sequenced *LHX1* and found three apparently pathogenic missense variants in 5/112 patients. Altogether, we identified either CNVs or variations in *TBX6* or *LHX1* in 30/112 (26.8%) MA patients. CNVs were found in 12/112 (10.7%), patients, novel variants in *TBX6* or *LHX1* in 7/112 (6.3%), and rare variants in *TBX6* in 15/112 (13.4%) patients. Furthermore, four of our patients (4/112, 3.6%) were shown to carry variants in both *TBX6* and *LHX1* or a CNV in combination with *TBX6* variants lending support to the complex genetic etiology of MA.

**Conclusions:**

We have identified *TBX6* as a new gene associated with MA. Our results also support the relevance of *LHX1* and CNVs in the development of this congenital malformation.

## Background

Müllerian aplasia (MA) is a rare disorder of the female reproductive tract presenting as congenital loss of a functional uterus and vagina. MA is commonly diagnosed in adolescent females due to lack of menstruation and has a large impact on a woman’s life. Inability of normal sex life prior to treatment and infertility combined with psychosocial problems make it one of the difficult fertility disorders diagnosed in young females. MA is also referred to as MURCS association (Müllerian duct aplasia, Renal dysplasia and Cervical Somite anomalies [MIM 601076]), because renal and skeletal malformations are associated with the disorder. Despite MA, the patients have a normal female karyotype and secondary sexual characteristics [1]. The incidence is estimated to be at least one in 5000 according to a population-based study in Finland [2].

During vertebrate embryogenesis, the female reproductive tract forms as a part of the urogenital system derived from the intermediate mesoderm of the developing embryo. The urogenital system encompasses the kidneys, gonads and the urinary and reproductive tracts. The female reproductive tract primarily develops from the Müllerian ducts (MD), which form as an invagination of the coelomic epithelium and further develop into the upper two-thirds of the vagina, the uterus and the Fallopian tubes [3,4].

Most patients with MA have the Mayer-Rokitansky-Küster-Hauser (MRKH [MIM 277000]) phenotype with presence of small remnants of the uterus and with unilateral/bilateral Fallopian tubes. A small group of patients are reported with complete loss of Müllerian derivatives, called total Müllerian aplasia [1,2].

The genetic background of MA has been intensively studied. The anti-Müllerian hormone (*AMH*), essential for MD regression during male differentiation, its receptor *AMHR2* and members of the *HOXA* and *WNT* gene families were primarily investigated in MA patients, but no mutations were found [5-12]. More recently mutations in wingless-type MMTV integration site family, member 4 (*WNT4)* and the LIM homeobox 1 *(LHX1)*, have been reported to be causative of MA [13-18]. However, *WNT4* defects occur in MA patients with hyperandrogenism not usually associated with the syndrome [13-16]. The *LHX1* defects were reported in patients with MRKH, the major clinical phenotype of MA, but only in two cases thus far [17,18]_._ The mutant mouse model for *LHX1* (*Lim1*−/−) lacks uterus and oviducts, but has normal ovaries coinciding with MA phenotype in humans [19]. Recent mutation screening efforts in a small number of MA patients in other genes involved in the development of the MD, namely *RARG*, *RXRA*[20], *CTNNB1*[21], *PBX1*[22], *PAX2*[23], *DLGH1* and *LAMC1*[24] have all been negative. The cause of MA is therefore still unknown for the vast majority of the patients.

By copy number analysis, several candidate regions have been implicated in MA [17,20,25-29]. Of these, most promising are regions on 16p11.2 and 17q12. The 17q12 region includes *LHX1*and another candidate gene, *HNF1B*. *HNF1B* has been associated with genital malformations, diabetes and renal cysts [30], but no mutations in MA patients have been identified to date [28], [18]. In the 16p11.2 region, deletions were recently reported in four patients with MA [29]. Deletions in this region have previously been associated with autism [31,32], developmental delay [33] and obesity [34], while duplications have been associated with autism [32], developmental delay [33] and schizophrenia [35]. The region includes approximately 26 genes with at least one gene with known function in mesodermal development, the T-box gene *TBX6*[36]. Here, we report results of CNV analysis and screening of *TBX6* and *LHX1* in 112 patients with MA, and suggest a role also for *TBX6* in the complex genetics of MA, and therefore in the development of the female reproductive system.

### Patients and methods

One-hundred and ten Finnish and two foreign patients originating from Russia and Middle East with a well-characterized MA phenotype were recruited to the study as previously described [37]. The patients had not been found to have symptoms or features of other syndromes. Two-hundred women with at least one normal pregnancy served as controls. Informed consent was obtained from all patients and controls before recruitment. The study protocol has been approved by the Ethics Committee of the Department of Obstetrics and Gynecology, Helsinki University Central Hospital, Finland, and the Finnish Ministry of Social Affairs and Health.

### Sample preparation

DNA from the patients and controls was extracted from peripheral blood samples using the Puregene DNA Isolation Kit (Gentra Systems, Minneapolis, MN, USA), or by the phenol-chloroform method. The quality and quantity of DNA was analyzed by NanoDrop ND-1000 spectrophotometer (Thermo Fisher Scientific, Waltham, MA, USA).

### Array Comparative Genomic Hybridization (aCGH)

Fifty Finnish patients, in whom detailed phenotypic data had been obtained by laparoscopy, were selected for the aCGH analysis, which was performed on a 180 K platform (Oxford Gene Technology, Yarnton*,* Oxford, UK). Briefly, samples were labeled with Cy3-and Cy5-deoxycytidine triphosphates by random priming. Purification, hybridization and washing were carried out as recommended by the manufacturer. Scanned images of the arrays were processed using Feature Extraction software (Agilent Technologies, Santa Clara, CA, USA). Genomic Workbench software (Agilent Technologies) was used for calling DNA copy number variations (CNVs) using the aberration detection statistical algorithm ADM-2, and sensitivity threshold 6.7. Duplication or deletion of a genomic segment was considered, when the log2 ratio of the Test/Reference intensities of a given region encompassing at least three probes was > 0.3 or < −0.3, respectively. Detected CNVs were compared to CNV data documented in the Database of Genomic Variants (DGV) [38].

### Multiplex ligation-dependent probe amplification (MLPA)

The MLPA method was used to study the 112 patients for larger deletions or duplications within *TBX6*. One hundred control samples were also investigated with the method. Synthetic MLPA probes (Additional file 1: Table S1) covering each of the coding exons of *TBX6* (exons 2–9, TBX6-001 transcript, Ensembl genome database hg19/GRCh37) [39], the non-coding exon 1, the 3’ UTR region and a presumptive *TBX6* promotor region upstream of exon 1 identified by CpG Plot [40] and CpG Island Searcher [41] were designed according to the manual Designing synthetic MLPA Probes (MRC-Holland, Amsterdam, the Netherlands). The specificity of the probe sequences were checked using the UCSC Genome Browser (GRCh37/hg19) [42], the RaW-Probe Version 0.15β program [43] and Mfold [44]. To enable even spreading of the probe sizes and compatibility with the P300 Human reference probemix (MRC-Holland), the lengths of nine of the eleven probes were adjusted using non-hybridizing stuffer fragments. The final sizes of the synthetic probes including the universal primers and a stuffer sequence were designed to range between 96 bp and 136 bp (IDT, San Jose, CA, USA).

The MLPA reactions were performed according to the manufacturer’s recommendations. In short, 200 ng of DNA was denatured and incubated with a mix including 1 μM of each of MLPA synthetic oligos and the P300 Human reference probemix for 16–18 h in 60°C. The probes were ligated to the DNA and amplified by PCR. Thereafter, the PCR products were visualized on an agarose gel (1.5%, Bioline, London, UK), appropriately diluted and combined with 1% formamide (Applied Biosystems) and GeneScan™-500 LIZ™ size standard (Applied Biosystems). The products were separated by capillary electrophoresis on an ABI3730XL DNA Analyzer (Applied Biosystems). The results were analyzed by GeneMapper software version 4.0 (Applied Biosystems) and verified by the following calculation. For each sample, each peak area was divided by the sum of all peak areas (derived from the 11 synthetic *TBX6* probe areas plus the 17 reference peak areas from the P300 Human reference probemix). Each quotient was further divided by the peak area of the reference sample. A result ≤0.7 or ≥1.3 was taken as suggestive for a deletion or duplication, respectively. All aberrant results were confirmed by a second independent MLPA analysis and deletions of the entire gene with SNP genotyping.

### Validation of aCGH and MLPA findings

High-throughput genotyping was performed using the Human Omni2.5-8 BeadChip v1.0 (Illumina, San Diego, CA, USA) capturing about 2.4 million markers to confirm the genomic alterations found by aCGH and MLPA. Data analyses were performed using GenomeStudio V2011.1 (Illumina). In addition, quantitative PCR (qPCR) was used to confirm six of the deletions and duplications detected by aCGH using the SYBR Green system (Roche Applied Science, Indianapolis, IN, USA) on a 7500 Fast Real-Time PCR System apparatus (Applied Biosystems, Foster City, CA, USA). We used six of the healthy Finnish female controls for copy number calibration, and the qPCR values for the *GAPDH* and *HPRT* for normalization. All samples were run in triplicate, and the data was analyzed with Microsoft Excel software (Microsoft Corp, Redmond, WA, USA) using the comparative ΔΔ^C^_t_ cycle threshold method (Applied Biosystems), which assumes that the calibrator DNA has two copies of the control genes.

### PCR and Sanger sequencing

The coding exons and exon-intron boundaries of *TBX6* (exons 2–9, TBX6-001 transcript, Ensemble genome database hg19/GRCh37) [39] were studied in 112 patients and 200 controls by Sanger sequencing. The PCR primers were designed using ExonPrimer [45] and Primer3 v. 0.4.0 [46]. The PCR was performed under standard conditions. The products were visualized by gel electrophoresis and subjected to sequencing in an ABI3730xl DNA Analyzer (Applied Biosystems) and analyzed by Sequencher 5.0 (Gene Codes, Ann Arbor, MI, USA). Likewise, all coding exons and exon-intron boundaries of *LHX1* (exons 1–5, LHX1-001 transcript, Ensemble genome database hg19/GRCh37) [39] were studied in the patients and 150 controls using primers designed by Primer3 v. 0.4.0 [46] and standard PCR reactions. Variants in the coding region of *LHX1* were sequenced in altogether 180 controls.

### RT-PCR

Peripheral blood was drawn from three patients and three controls using the PAXgene Blood RNA Tube (PreAnalytiX Gmbh, Hombrechtikon, Switzerland) and RNA was subsequently extracted by the PAXGene RNA Kit (Qiagen, Hilden, Germany) according to the recommended protocol. The extracted RNA was further treated with DNA-free kit (Applied Biosystems) to remove genomic DNA (gDNA). cDNA synthesis was done using High capacity cDNA reverse transcriptase kit (Applied Biosystems) in a final volume of 20 μl according to the manufacturer’s recommendations. RT-PCR was performed using DreamTaq PCR master mix (2x) (Thermo Scientific, Walthem, MA, USA), primers designed for exon 4 (5’ TACATTCACCCCGACTCTCC 3’) and exon 6 (5’ TGGCTGCAATCTTCAGTTGT 3’) by Primer3 v. 0.4.0 [46] and a touchdown thermocycling PCR program with annealing temperatures from 67°C to 55°C and the products were visualized by gel electrophoresis.

### Statistics

Differences in SNP allele frequency between patients and controls were calculated using the non-parametric Mann–Whitney-U test (PASW Statistics 18, SPSS, Chicago, IL, USA) using Bonferroni correction for multiple testing.

### Prediction programs

*In silico* analyses of genomic variants were performed using MutationTaster [47], PON-P [48] including PhD-SNP 2.0.6, PolyPhen 2.0.22, SIFT 4.0.3, SNAP 1.0.8 and I-Mutant 3.0.6 programs used for analysis, SpliceMan [49], SplicePort [50] and Human Splicing Finder [51] software programs.

## Results

Initially, we chose 50 patients for copy number analysis using aCGH and identified nine CNVs in eight (16%) of them (Table 1). Two of the identified CNVs have been previously reported in MA, namely deletions on 16p11.2 [29] and 17q12 [27-29], and we therefore decided to further investigate two interesting MA candidate genes within these two regions, namely *TBX6* and *LHX1*.

**Table 1:** Summary of aCGH results

**Locus**	**Patient ID**	**Size**	**CNV**	**Location**	**Genes within CNV**	**Confirmation method**
5p14.3	28	1.6 Mb	Del	5:18822021-20417776	CDH18	SNP array, qPCR
9q21.13	3	95 Kb	Del	9:74296070-74391713	TMEM2	SNP array
11q13.4	2	54 Kb	Del	11:73584463-73638725	CHCHD8, PAAF1	SNP array, qPCR
15q26.1	4	96 Kb	Del	15:90883372-90979449	ZNF774, IQGAP1	SNP array, qPCR
16p11.2	69	0.53 Mb	Del	16:29656457–30190734	SPN, QPRT, C16orf54, MAZ, PRRT2, C16orf53, MVP, CDIPT, LOC440356, SEZ6L2, ASPHD1, KCTD13, TMEM219, TAOK2, HIRIP3, INO80E, DOC2A, C16orf92, FAM57B, ALDOA, PPP4C, TBX6, YPEL3, GDPD3, MAPK3, LOC100271831	SNP array
16p13.3	42	143 Kb	Del	16:6213403-6356820	A2BP1	SNP array, qPCR
17q12	24	1.7 Mb	Del	17:31584620-33353268^a^	TBC1D3C, CCL3L1, CCL3L3, CCL4L2, CCL4L1, TBC1D3H, TBC1D3C, TBC1D3G, ZNHIT3, MYO19, PIGW, GGNBP2, DHRS11, MRM1, LHX1, AATF, ACACA, C17orf78, TADA2L, DUSP14, AP1GBP1, DDX52, HNF1B, LOC284100	SNP array
19q13.11^b^	49	194 Kb	Dupl	19:33532490-33727077	RHPN2, GPATCH1, WDR88, LRP3, SLC7A10	SNP array, qPCR
19q13.12^b^	49	0.6 Mb	Dupl	19:35731695-36309487	LSR, USF2, HAMP, MAG, CD22, FFAR1, FFAR3, FFAR2, KRTDAP, DMKN, SBSN, GAPDHS, TMEM147, ATP4A, HAUS5, RBM42, ETV2, COX6B1, UPK1A, ZBTB32, MLL4, TMEM149, U2AF1L4, PSENEN, LIN37, HSPB6, C19orf55, SNX26, PRODH2	SNP array, qPCR

^a^location according to UCSC genome database hg18.

^b^likely to include non-duplicated regions, possibly due to other chromosomal rearrangements e.g. inversions within the region or non-functional aCGH probes.

CNV = copy number variations, Del = deletion, Dupl = duplication.

Location according to UCSC genome database hg19 [42].

**Figure 1:**
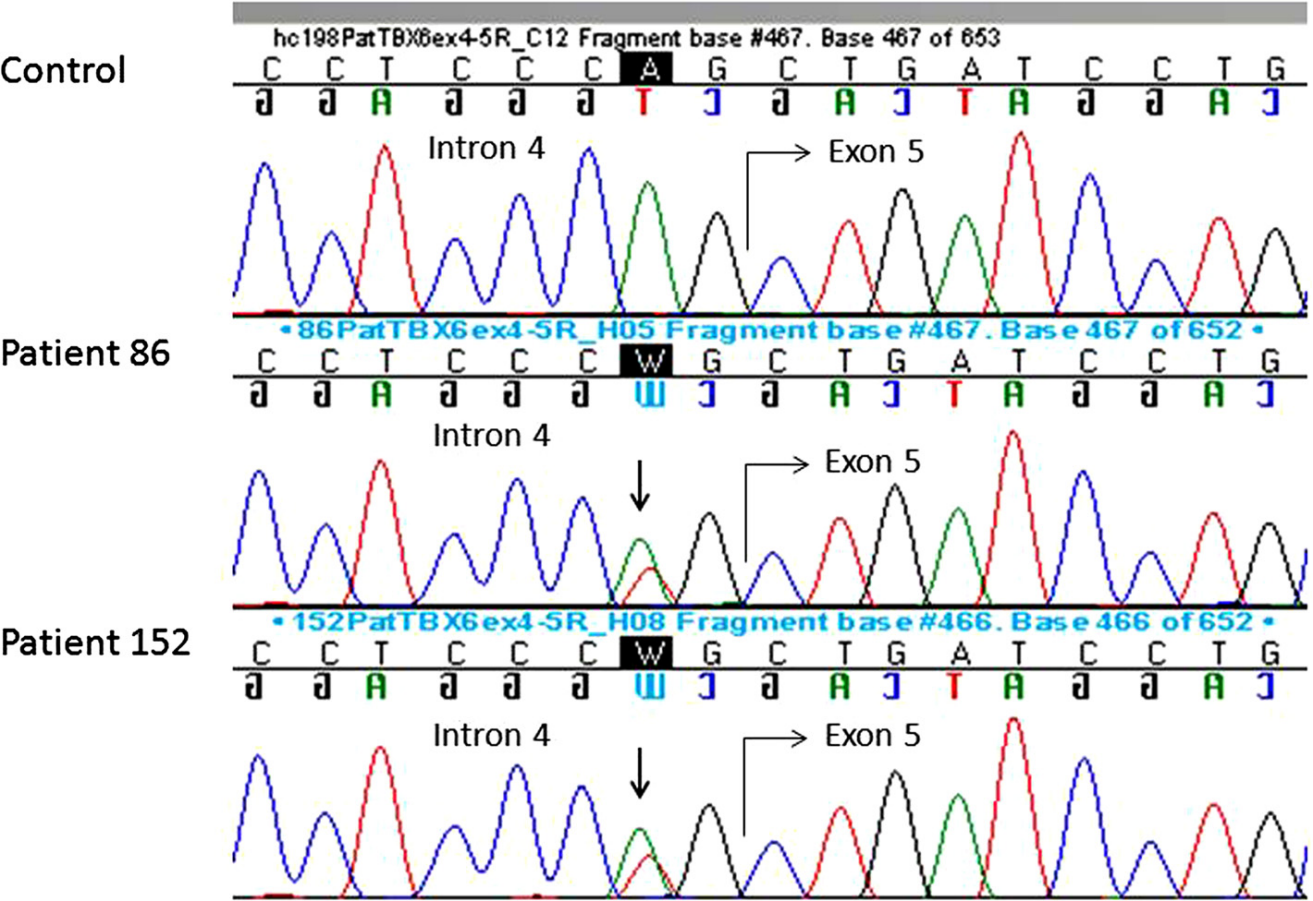
**Novel splice site mutation of *TBX6 *in two MA patients.** Splice site mutation c.622-2A>T (g.30100162 T>A) in two MA patients compared to healthy control sample (on the top). The mutation is indicated by an arrow.

DNA from 112 MA patients was Sanger sequenced for the entire coding region and the exon-intron boundaries of *TBX6* (exons 2–9, RefSeq NM_004608.3). We found a novel heterozygous splice site mutation c.622-2A>T (g.30100162 T>A) in two patients (Figure 1). The mutation was not found by sequencing 200 Finnish healthy females, in dbSNP [52], in 1000 Genomes database [53], or in cohort data comprising of 1532 alleles of Finnish ancestry (A-P Sarin and A. Palotie, personal communication). In Exome Variant Server (EVS, [February, 2013 accessed]) [54], where >12000 alleles of African American and European American ancestry have been sequenced for *TBX6*, no allele with this variant was reported.

**Figure 2:**
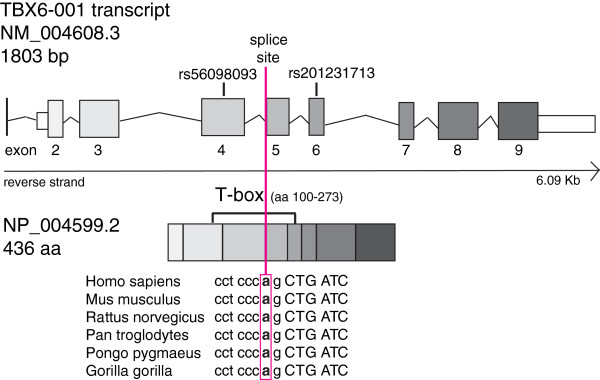
**Schematic view of *TBX6.*** A schematic view of *TBX6* on genomic and protein level with indication of rare variants (rs56098093 and rs201231713) and the splice site mutation site (g.30100162 T>A, c.622-2A>T in violet brackets and a line) and its conservation among species.

The mutation (c.622-2A>T) is situated in the canonical sequence of the highly conserved splice acceptor site (AG) for exon 5 (Figure 2). MutationTaster [47], SplicePort [50] and Human Splicing Finder [51] prediction programs all indicate that the mutation causes loss of the functional acceptor site while the SpliceMan predictor program [49] gives a prediction score of 69% for the likelihood of the mutation to disrupt splicing. The loss of the splice acceptor site is likely to result in skipping of exon 5 or the use of cryptic splice sites. The c.622-2A>T mutation resides within the highly conserved DNA-binding domain, the T-box, of the protein (aa 100–273, encoded from the 3’end of exon 3 to the 3’ end of exon 6) (Figure 2). RT-PCR revealed presence of at least three different transcripts ranging from about 270 to 480 kb in size, all present in both patient and control samples.

In addition, we found six *TBX6* variants, all previously reported in dbSNP [52] (Table 2). Noteworthy is that the exonic missense variants in exon 4 (rs56098093; g.30100401C>T; p.Gly162Ser) and exon 6 (rs201231713; g.30099890C>T; p.Arg272Gln) were found in a higher frequency in patients compared to healthy females. The minor allele frequency (MAF) for rs56098093 was 8.0% for the patients and 2% for the controls and MAF for rs201231713 was 5.8% for the patients and 2% for the controls. The differences were statistically significant (*P*-value 0.0021 for rs56098093 and 0.0002 for rs201231713), after a stringent Bonferroni correction for multiple testing (*P*-value / 2 < 0.05). Interestingly, two Finnish MA patients were homozygous for both variants (AA and AA, respectively) while none of the 200 sequenced controls were homozygous for either of the variants. In the Finnish cohort data of 1532 alleles, the frequencies of the variants were 0.5% (exon 4) and 0.7% (exon 6), respectively (A-P Sarin and A. Palotie, personal communication). Both variants are situated within the highly conserved DNA-binding T-box region of the gene (Figure 2). MutationTaster [47] predicts both variants as disease causing with loss of the DNA-binding region of the gene. PON-P [48] predicts both variants as pathogenic with a probability of pathogenicity scores of 0.81 (Gly162Ser) and 1 (Arg272Gln), respectively.

**Table 2:** **Variants of *TBX6 *with allele frequencies**

**Variant**^**a**^	**rs number**^**b**^	**Location in *TBX6***	**Genotype of patients**^**c**^	**Genotype of controls**^**c**^
**Predicted change**			**N=112**	**N=200**
g.30102391G>A	rs112565029	intron 2	CC: 107 (95.5%)	CC: 187 (93.5%)
c.118+6C>T			CT: 5 (4.5%)	CT: 13 (6.5%)
			TT: 0 (0%)	TT: 0 (0%)
g.30100402G>A	rs147485102	exon 4	CC: 111 (99.1%)	CC: 198 (99%)
p.Ser161=			CT: 1 (0.9%)	CT: 2 (1%)
			TT: 0 (0%)	TT: 0 (0%)
**g.30100401C>T**	**rs56098093**	**exon 4**	**GG: 97 (86.6%)**	**GG: 192 (96%)**
**p.Gly162Ser**			**GA: 12 (10.7%)**	**GA: 8 (4%)**
			**AA: 3 (2.7%)**	**AA: 0 (0%)**
**g.30100162 T>A**	**-**	**intron 4**	**AA: 11 (98.2%)**	**AA: 200 (100%)**
**c.622-2A>T**			**AT: 2 (1.8%)**	**AT: 0 (0%)**
			**TT: 0 (0%)**	**TT: 0 (0%)**
**g.30099890C>T**	**rs201231713**	**exon 6**	**GG: 101 (90.2%)**	**GG: 198 (99%)**
**p.Arg272Gln**			**GA: 9 (8.0%)**	**GA: 2 (1%)**
			**AA: 2 (1.8%)**	**AA: 0 (0%)**
g.30098022G>A	rs200310768	intron 7	CC: 109 (97.3%)	CC: 194 (97%)
c.914-6C>T			CT: 3 (2.7%)	CT: 6 (3%)
			TT: 0 (0%)	TT: 0 (0%)
g.30097630C>T	rs2289292	exon 9	GG: 32 (28.6%)	GG: 67 (33.5%)
p.Pro409=			GA: 55 (49.1%)	GA: 95 (47.5%)
			AA: 25 (22.3%)	AA: 38 (19%)

The splice site mutation and the rare variants indicated in bold.

^a^variations presented according to genomic reference sequence (g.) NC_000016.9, coding DNA reference sequence (c.) NM_004608.3 and/or protein reference sequence (p.) NP_004599.2, Genome Build 37.3, dbSNP [52].

^b^rs numbers for previously known SNPs according to dbSNP [52].

^c^number of genotypes reported as: reference allele/reference allele; reference allele/alternative allele; alternative allele/alternative allele.

**Figure 3:**
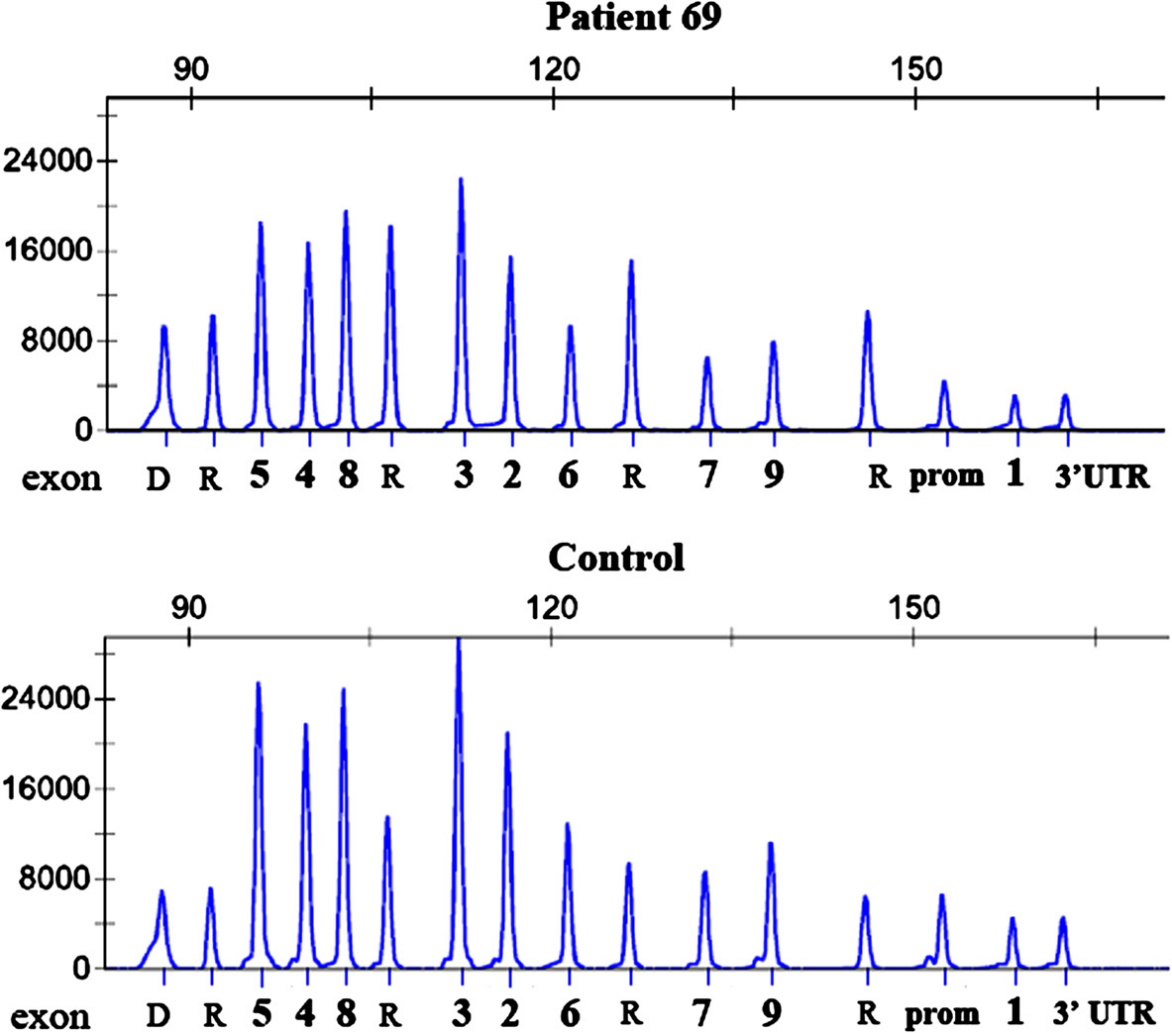
**Detection of *TBX6 *deletions by multiplex ligation probe amplification (MLPA).** A ~30% reduction in peak size of the *TBX6*-specific probes in patient (above) compared to control sample (below). Each exon of *TBX6* is indicated by its corresponding number (in bold), prom = promotor, 3’UTR = 3’untranscribed region, D =a quality control fragment, and R=four reference probes.

**Figure 4:**
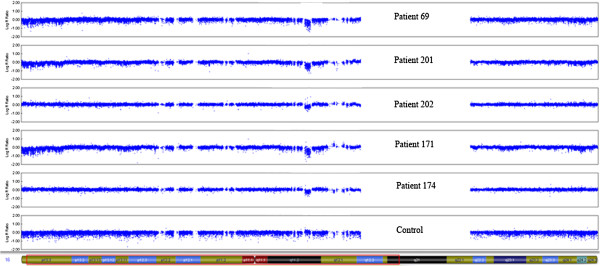
**SNP array genotyping confirming the 16p11.2 deletion.** Five MA patients were confirmed with the 16p11.2 deletion using HumanOmni2.5-8 v1.0, Illumina. The patient samples are here shown together with a normal control sample (Genome Viewer, GenomeStudio V2011.1, Illumina).

MLPA analysis of *TBX6* showed heterozygous deletion of the entire *TBX6* in five out of 112 (4.5%) patients, including the patient with 16p11.2 deletion found by aCGH (Figure 3). The peak area-based calculations indicated that these patients have a loss of one copy for all eleven *TBX6* probes (value approximately 0.7) compared to the reference with two gene copies (value 1.0) and to the 100 healthy female controls. To delineate further the nine CNVs found by aCGH and the *TBX6* deletions found by MLPA, these patients were also studied by SNP genotyping (HumanOmni2.5-8, Illumina). The heterozygous *TBX6* deletions found by MLPA were shown to embrace not only *TBX6*, but the same 0.53 Kb deletion on 16p11.2, which was initially detected by aCGH in patient 69, in all five patients (Figure 4).

**Figure 5:**
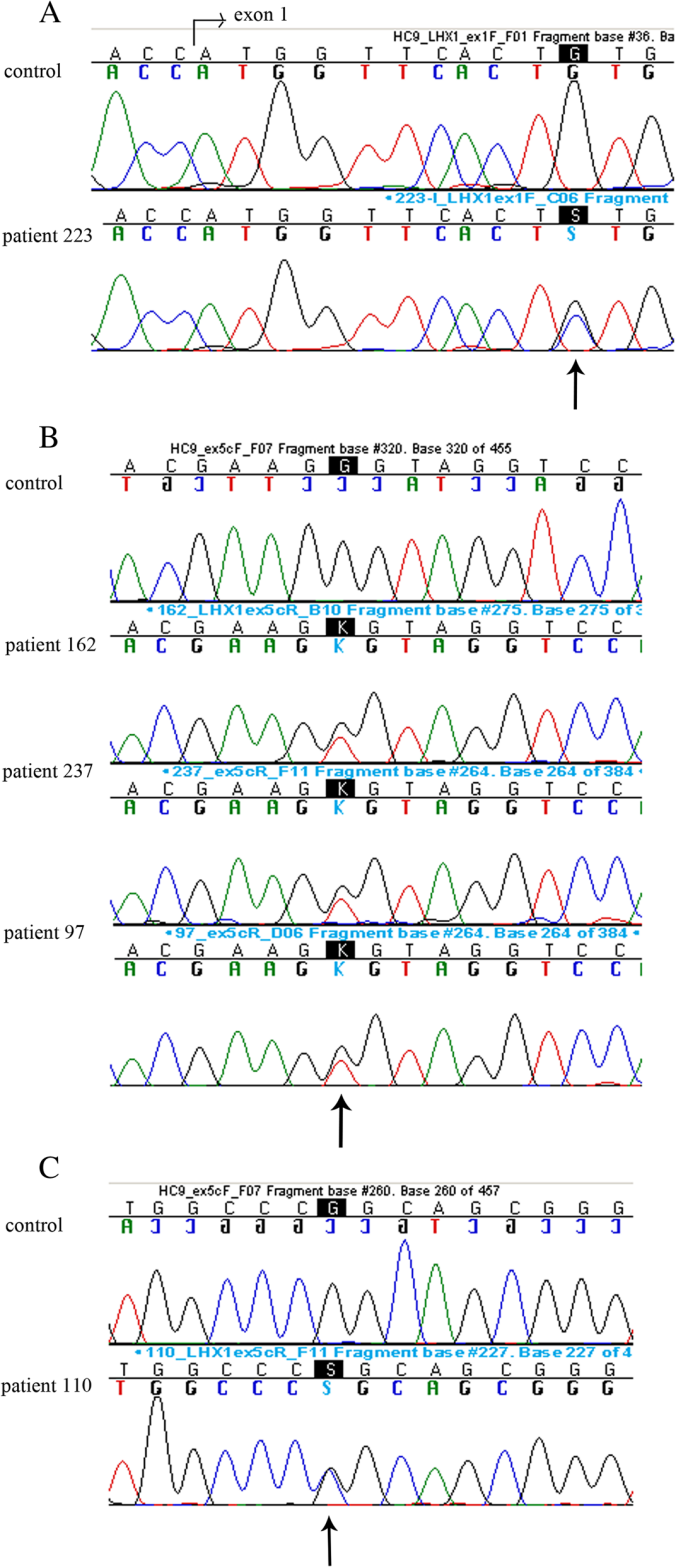
**Three novel *LHX1 *variants found in five MA patients.** Three novel *LHX1* missense variants found in five MA patients. **A)** The g.35295505G>C (p.Cys4Ser) variant in control and patient 223, **B)** the g.35300142C>A (p.Pro312His) in control and patients 162, 237, and 97 and **C)** the g.35300202C>G (p.Arg332Pro) in control and patient 110. The variations are indicated by arrows.

By sequencing the entire coding region and the exon-intron boundaries of *LHX1*, we found altogether five patients with novel variants in the gene (Figure 5). One patient had a missense variant (g.35295505G>C; p.Cys4Ser) in exon 1, three patients had the same missense variant in ex 5 (g.35300142C>A; p.Pro312His) and one patient had another missense variant in ex 5 (g.35300202C>G; p.Arg332Pro) (Table 3). None of the variants are reported in dbSNP [52], 1000 Genomes database [53] or EVS, except Arg332Pro, which was found in 1/12783 alleles in EVS. MutationTaster [47] predicts all three missense variants as disease causing. The program predicts the Cys4Ser variant to cause loss of the LIM zinc-binding 1 domain (aa 4–54) of the gene. PON-P [48] predicts Cys4Ser as unclassified with a probability of pathogenicity score 0.75, Pro312His and Arg332Pro as neutral with probability of pathogenicity scores of 0.03 and 0.01, respectively.

**Table 3:** **Variants of *LHX1 *with allele frequencies**

**Variant**^**a**^	**rs number**^**b**^	**Location in *LHX1***	**Genotype of patients**^**d**^	**Genotype of controls**^**d**^
**Predicted change**			**N=112**	**N= 180**
g.35295505G>C	-	exon 1	GG: 111 (99.1%)	GG: 180 (100%)
p.Cys4Ser			GC: 1 (0.9%)	GC: 0 (0%)
			CC: 0 (0%)	CC: 0 (0%)
g.35300142C>A	-	exon 5	CC: 109 (97.3%)	CC: 180 (100%)
p.Pro312His			CA: 3 (2.7%)	CA: 0 (0%)
			AA: 0 (0%)	AA: 0 (0%)
g.35300202C>G	TMP_ESP_17_35300202^c^	exon 5	CC: 111 (99.1%)	CC: 180 (100%)
p.Arg332Pro			CG: 1 (0.9%)	CG: 0 (0%)
			GG: 0 (0%)	GG: 0 (0%)

^a^variations presented according to genomic reference sequence (g.) NC_000017.10, coding DNA reference sequence (c.) NM_005568.3 and/or protein reference sequence (p.) NP_005559.2, Genome Build 37.3, dbSNP [52].

^b^rs numbers for previously known SNPs according to dbSNP [52] or Exome Variant Server (EVS) [54].

^c^one allele of 12783 reported in EVS [54].

^d^number of genotypes reported as: reference allele/reference allele; reference allele/alternative allele; alternative allele/alternative allele.

**Figure 6:**
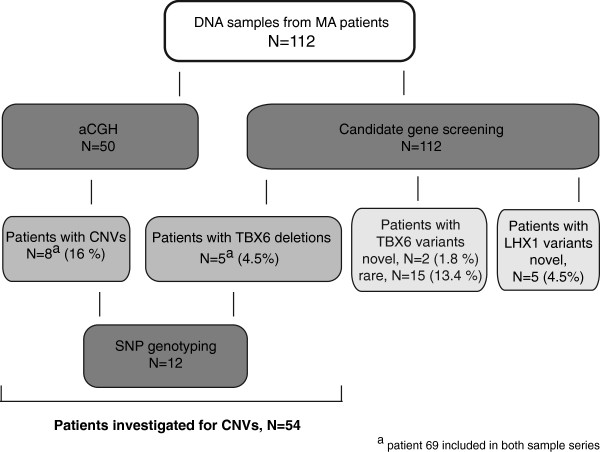
**Flow chart summarizing main results and methods.** A flow chart summarizing the main results and methods used in the study, including number of patient samples investigated by each of the methods.

A flow chart summarizing the main results and methods is shown in Figure 6.

## Discussion

We report here the results of genetic studies on Müllerian aplasia, namely, results of *TBX6* and *LHX1* mutation screening in 112 MA patients and CNV analysis in a subset of them.

Sequencing of *TBX6* (located in 16p11.2) revealed a splice mutation in two patients and rare missense variants in 15 patients (13.4%). *TBX6* is a transcription factor that functions in early embryogenesis. It resides on the minus strand with a full-length transcript of 1806 bp, encoding a 436 aminoacid protein (NP_004599.2). *TBX6* is a member of a phylogenetically well-conserved T-box gene family, where all members share the similar N-terminal DNA-binding domain, the T-box [55]. We identified a c.622-2A>T splice site mutation in two patients (one with MRKH and the other with total MA and with ovarian aplasia) and a rare homozygous missense variant in exon 4 and exon 6 in two patients. The splice site mutation is situated in the highly conserved splice acceptor site (AG) of exon 5. According to the *in silico* prediction programs, the mutation is likely to decrease correct splicing of exon 5, which is located in the highly conserved DNA-binding T-box, or result in use of cryptic splice sites. To investigate the consequences of this mutation on mRNA level, we extracted RNA from whole blood, which was available from one of the patients with the mutation. Several PCR products were obtained, indicating that several mRNA transcripts are present, but the sizes of the products did not differ in patient and control samples. The reason for this is most likely that very little or none of the mutated transcript is transcribed. Two patients were homozygous for both the exon 4 and the exon 6 variant of *TBX6*, ten patients were heterozygous for both, and three patients had the exon 4 variant in a heterozygous state. Both variants are situated within the conserved T-box region and the prediction programs indicate that the variants have an effect also on the protein level. We therefore believe that these variants together with other variations have a role in the multifactorial etiology of MA. However, to finally determine the nature of the *TBX6* splice site mutation and of the rare homozygous variants in ex 4 and 6 of the gene, *in vitro* functional studies are needed.

*TBX6* has been suggested to associate with congenital scoliosis in the Chinese Han population [56]. Interestingly, vertebral changes giving rise to e.g. scoliosis, are commonly found in patients with MA. Recently, a missense mutation in the last codon of the *TBX6* transcript was observed in a Macedonian family with the autosomal dominant form of spondylocostal dysostosis (SCD), characterized by segmentation defects of the vertebrae [57]. The affected individuals were all males and therefore there is no data about the effect of the *TBX6* mutation on the development of the female urogenital tract. The family had also experienced six infant deaths (one girl, five boys) of unknown cause without further phenotype data [57,58]. Mouse knock-out studies have shown that *Tbx6* is important for segmentation of the somites in the paraxial mesoderm [59] and for left/right patterning [60]. White and coworkers have shown that *Tbx6* interacts with the Notch-ligand delta-like 1 (*Dll1*) [36], a component in the Notch pathway. Also WNT signaling, acting upstream of *Tbx6*, has been shown to regulate *Dll1* activity [61]. Both Notch and WNT signalling are import in the so called segmentation clock, cyclic expression of genes under which each somite is formed from the presomitic mesoderm [62]. It is conceivable that a similar segmentation clock could operate in the segmentation of Müllerian ducts to form upper vagina, uterus and Fallopian tubes.

A spontaneous mouse model for *Tbx6* is the homozygous Tbx6^rv^ (rib-vertebrae). It is caused by an insertion in the *Tbx6* promotor upstream of the transcriptional start site resulting in a hypomorphic allele and reduced transcript expression [63]. The mutation affects somite formation and patterning featured as malformations of the axial skeleton and fusions of adjacent segments. Interestingly, the model also shows malformations in the renal and urinary system e.g. with reported single kidney and in the reproductive system with reduced female fertility. Unfortunately, no detailed information is available on the status of the reproductive tract or on why the female mice are reported as poor breeders [64]. However, the Tbx6^rv^ has phenotypic similarities with MURCS, as the phenotype includes skeletal and renal malformations. In addition, if the reduced fertility is caused by defects in the reproductive tract, the mouse model would be of interest for functional studies in MA.

Previous candidate gene studies of *LHX1* residing in the 17q12 deletion region revealed two MA patients with heterozygous mutations [17,18]. The gene had been suggested as a strong candidate for mutation screening based on studies in mice [19] showing that *lim1*, which is 99.5% homologous to the human *LHX1* gene [65], is expressed in the epithelium of the developing Müllerian duct during its formation. Female lim1-null mice present normal ovaries but completely lack all derivatives of the Müllerian ducts (oviducts, uterus, cervix and the upper region of the vagina), thereby resembling the total MA phenotype. Therefore, we performed sequencing of *LHX1* in our patient cohort and found three novel missense variants in five MA patients (four Finnish and one foreign). The *in silico* prediction programs indicate that the exon 1 variant is deleterious, while the data for the other two are discrepant. Further studies are needed to define their role in the development of MA. The patient with the exon 1 variant (Cys4Ser) had total MA. Interestingly, one patient with the exon 5 variant (Pro312His) was also homozygous for both rare *TBX6* variants (rs56098093 and rs201231713). Another patient with the same *LHX1* exon 5 variant was heterozygous for both rare *TBX6* variants. Furthermore, the other patient homozygous for both rare *TBX6* variants was also identified with a 11q13.4 deletion.

We also identified nine different CNVs in 12 patients (10.7%) using aCGH and SNP genotyping. Seven of the CNVs are novel (five deletions found in one patient each and two duplications found in the same patient) reported here for the first time in association with MA, and have not been documented in normal controls (DGV) [38]. Nevertheless, we cannot conclude if they have a role in the development of MA. However, the genes located within these CNVs are possible candidate genes for further studies. Two CNVs, namely deletions in 16p11.2 and 17q12, have previously been described in four [29] and eight [17,27-29] MA patients, respectively. We found the 16p11.2 deletion in five patients and the 17q12 deletion in one patient, adding the total number of MA patients to nine for both deletions. Although the number of patients identified with the deletions is low, they have to date been identified in MA patients originating in two and five different populations, respectively, and therefore they are likely to be relevant for the development of MA.

The 16p11.2 region is prone to rearrangements and CNVs in the region have previously been associated with different clinical phenotypes [31-33,66], [35], [34]. Bijlsma and coworkers sequenced the coding regions of A*LDOA*, *TBX6* and *SPN* in four patients with 16p11.2 deletion and mild mental retardation. No mutations were found and they concluded that CNVs of 16p11.2 are associated with variable phenotypes [67]. In our study, five MA patients showed the deletion. Three of these had skeletal findings including scoliosis and minor vertebral defects, which are known symptoms of MA. In addition, one patient had an unexplained weight gain of 32 kg within 2 years and another had experienced seizures of unknown cause. Because the 16p11.2 region is large and includes many genes, it is reasonable to assume that several phenotypes, including MA, may be associated with this deletion.

## Conclusions

Taken together, our findings suggest that both *TBX6* and *LHX1* participate in the control of the development of the female reproductive tract. While the number of mutations so far identified in these two genes is low, more mutations are likely to be found when extending the mutation screening to other populations and to embrace the promotor regions, intronic sequences and the 5’ and 3’ UTR regions of the genes. Our results also strengthen the previous finding that deletions in 16p11.2 and 17q12 are associated with MA. Furthermore, the results support the hypothesis that MA indeed is a complex trait as demonstrated by the finding that four of our patients were shown to carry variants in both *TBX6* and *LHX1* or a CNV in combination with *TBX6* variants. While our findings on *TBX6* need to be confirmed in independent patient cohorts, it is equally important that further genes in the deleted regions are studied for an association with MA. Likewise, functional studies of the identified genes will enhance the understanding of how the Müllerian derivatives are formed during normal embryogenesis and how and why the development is disrupted to result in Müllerian agenesis, the congenital abnormality, which so profoundly affects the life of young females.

## Competing interests

The authors declare that they have no competing interests.

## Authors’ contributions

MS designed and performed the majority of the experiments, performed data analysis and wrote the manuscript. EF designed and performed aCGH, analyzed the data and contributed to writing the manuscript. MH, VJ and LMP contributed to recruitment and examination of patients, clinical data collection, and critically revised the manuscript. CR analyzed aCGH data and contributed in writing the manuscript. HL and KA designed the study and contributed in writing the manuscript. KA is the guarantor of the study. All authors have seen and approved the final version.

## Supplementary Material

Additional file 1: Table S1 Synthetic MLPA probes designed for *TBX6*. One probe consists of two oligonucleotides: the Left Probe Oligonucleotide (LPO) and the Right Probe Oligonucleotide (RPO) with optional stuffers (small cases). On the 5’end of LPO is the Forward PCR primer (all caps, bold) and on the 3’end is the Left Hybridizing Probe (LHS, all caps). On the 5’end of RPO is the Right Hybridizing Probe (RHS, all caps) and on the 3’ end the binding sequence of the Reverse PCR primer (all caps, bold). The RPOs are 5’phosphorylated. (The file is attached as a Word-document and named: Additional file 1 Sandbacka). Click here for file
