# Supplementary material for: TBX6, LHX1 and copy number variations in the complex genetics of Müllerian aplasia
Source: Orphanet J Rare Dis. 2013 Aug 16;8:125. doi: 10.1186/1750-1172-8-125 (PMC3847609; doi:10.1186/1750-1172-8-125)
Supplement: Additional file 1: Table S1 — Synthetic MLPA probes designed for TBX6. One probe consists of two oligonucleotides: the Left Probe Oligonucleotide (LPO) and the Right Probe Oligonucleotide (RPO) with optional stuffers (small cases). On the 5’end of LPO is the Forward PCR primer (all caps, bold) and on the 3’end is the Left Hybridizing Probe (LHS, all caps). On the 5’end of RPO is the Right Hybridizing Probe (RHS, all caps) and on the 3’ end the binding sequence of the Reverse PCR primer (all caps, bold). The RPOs are 5’phosphorylated. (The file is attached as a Word-document and named: Additional file 1 Sandbacka). [file 1750-1172-8-125-S1.doc]

**Additional file 1: Synthetic MLPA probes designed for TBX6.**

One probe consists of two oligonucleotides: the Left Probe Oligonucleotide (LPO) and the Right Probe Oligonucleotide (RPO) with optional stuffers (small cases). On the 5’end of LPO is the Forward PCR primer (all caps, bold) and on the 3’end is the Left Hybridizing Probe (LHS, all caps). On the 5’end of RPO is the Right Hybridizing Probe (RHS, all caps) and on the 3’ end the binding sequence of the Reverse PCR primer (all caps, bold). The RPOs are 5’phosphorylated.

| Probe | Sequence (5’ to 3’ direction) | Length (bp) | Total length (bp) of probe (LPO+RPO) |
| --- | --- | --- | --- |
| TBX6_promoter_LPO | **GGGTTCCCTAAGGGTTGGA**cctaaagccc agccaatcacctaaagtCGTGTAATCCGGGAAGGGGGAGCTCAAGC | 75 | 153 |
| TBX6_promoter_RPO | **5’Phos**:ggagatttccgcccctatagacctgtgcgtgaaaggtgatgcggtctaacgaaac**TCTAGATTGGATCTTGCTGGCAC** | 78 |
| TBX6_ex1_LPO | **GGGTTCCCTAAGGGTTGGA**cctaaagtcaaccttcggttgaccttgagggttCGTCAGAAGCTGTCGGACTGTGAGCG | 78 | 158 |
| TBX6_ex1_RPO | **5’Phos**:CCTTCGAACTTTGGAGGCTTGGCTCGcacactgaaaggtgatgcggtctaacgaaac**TCTAGATTGGATCTTGCTGGCAC** | 80 |
| TBX6_ex2_LPO | **GGGTTCCCTAAGGGTTGGA**gtgtacggacGCGAGACCTGAGGCCAGACGGAACTACAA | 58 | 119 |
| TBX6_ex2_RPO | **5’Phos**:CATGTACCATCCACGAGAATTGTACCCGTtaacgaaac**TCTAGATTGGATCTTGCTGGCAC** | 61 |
| TBX6_ex3_LPO | **GGGTTCCCTAAGGGTTGGA**tacggacctaaagtTGAGCCTGGAGAACCGGGAGCTAT | 57 | 114 |
| TBX6_ex3_RPO | **5’Phos**:GGAAGGAGTTCAGCTCTGTGGGAActaacgaaac**TCTAGATTGGATCTTGCTGGCAC** | 57 |
| TBX6_ex4_LPO | **GGGTTCCCTAAGGGTTGGA**TGGACCCCGAGGCCCGCTACTTGTTTCTT | 48 | 100 |
| TBX6_ex4_RPO | **5’Phos**:CTGGATGTGATTCCGGTGGATGGGGCTCG**TCTAGATTGGATCTTGCTGGCAC** | 52 |
| TBX6_ex5_LPO | **GGGTTCCCTAAGGGTTGGA**tcTGGCCTCCTTCCGCTTCCCCGAGACCA | 48 | 98 |
| TBX6_ex5_RPO | **5’Phos**:CATTCATCTCCGTGACAGCCTACCAGA**TCTAGATTGGATCTTGCTGGCAC** | 50 |
| TBX6_ex6_LPO | **GGGTTCCCTAAGGGTTGGA**gtgtacggacctaaCACACAACTGAAGATTGCAGCCAATCCCT | 62 | 124 |
| TBX6_ex6_RPO | **5’Phos**:TTGCCAAAGGCTTCCGGGAGAACGGCAGAtgagtgcatg**TCTAGATTGGATCTTGCTGGCAC** | 62 |
| TBX6_ex7_LPO | **GGGTTCCCTAAGGGTTGGA**gtgtacggacctaaagttcccCCCAGGGAGCGAGACGCCCGTGTGAA | 66 | 135 |
| TBX6_ex7_RPO | **5’Phos**:GAGGAAACTGCGGGGCCCAGAGCCAGgtgatgcggtctaacgaaac**TCTAGATTGGATCTTGCTGGCAC** | 69 |
| TBX6_ex8_LPO | **GGGTTCCCTAAGGGTTGGA**tacggacctaaCACCTCTGTGTGGTGGCCCCA | 51 | 104 |
| TBX6_ex8_RPO | **5’Phos**:GTGCTGAGGCCTACCTCCTGCgagtgcatg**TCTAGATTGGATCTTGCTGGCAC** | 53 |
| TBX6_ex9_LPO | **GGGTTCCCTAAGGGTTGGA**cagtgtacggacctaaagttccCACTTTCTCCAAGGGGGCCCCTTCCCTCTAC | 72 | 140 |
| TBX6_ex9_RPO | **5’Phos**:CATACACCGCGCCTGGGGGCTATCTGGATGTcggtctaacgaaac**TCTAGATTGGATCTTGCTGGCAC** | 68 |
| TBX6_3’UTR_LPO | **GGGTTCCCTAAGGGTTGGA**cctaaagtcaaccttcggttgaccttgagggttccctaCCACACACACTGGTGCAGGCCACAC | 82 | 163 |
| TBX6_3’UTR_RPO | **5’Phos**:CAGTCTGTTGTTCTGGGACCAGAGTaataactgcacgaggtaacacaagatggctatg**TCTAGATTGGATCTTGCTGGCAC** | 81 |
